# Monthly eDNA Monitoring of an Invasive Bryozoan, *Bugulina californica*, in Seawater Using Species-Specific Markers

**DOI:** 10.3390/ani11071966

**Published:** 2021-06-30

**Authors:** Philjae Kim, Tae-Joong Yoon, Sook Shin

**Affiliations:** 1 Institute of Marine Biological Resources, Sahmyook University, Seoul 01795, Korea; swubio@naver.com (P.K.); redyoon@gmail.com (T.-J.Y.); 2 Division of Ecological Conservation, Bureau of Ecological Research, National Institute of Ecology, Seocheon-gun 33657, Korea; 3 Department of Animal Biotechnology & Resource, Sahmyook University, Seoul 01795, Korea

**Keywords:** marine invasive species, environmental DNA, real-time PCR, *Bugulina californica*, biomass monitoring

## Abstract

**Simple Summary:**

*Bugulina californica*, marine invasive bryozoan, is hard to monitor the biomass and presence because of their habitat in underwater. Additionally, they have life stage difficult to find such as larva, and we need an effective survey method to detect whole life stages for monitoring early invasion stage. Therefore, we tried to applied environmental DNA to monitor the monthly changes of *B. californica* in harbors of Korea. We collect seawater environmental samples and developed a molecular target species detection method to detect *B. californica* DNA of monthly changes. We analyzed the environmental samples using our molecular markers and calculated the DNA copies. We determined method of environmental DNA assay as effectiveness survey technique for marine invasive species which has a non-visual life stage and spatial changes of whole biomass.

**Abstract:**

Environmental DNA (eDNA) method used by many ecologists as effective investigation tool can detect endangered species, rare species, and invasive species. In case of invasive species, eDNA method help to monitor the target species when the species was hard to detect through the traditional survey such as the early stage of invasion, low abundance, and larva or juvenile stage. The bryozoan, *Bugulina californica*, was known as a marine fouling invasive species in Korea since its first reported in 1978. This species expanded nationwide, and damages to ascidian aquaculture through attached on the ship hulls and artificial facilities. To monitor the distribution and biomass of invasive bryozoan, *B. californica*, the qPCR analysis of environmental DNA was performed on seawater samples from 12 harbors. In this study, we designed species-specific markers which can calculate the detected DNA copies of *B. californica*, and the presence and monitoring of this species can be more accurately estimated by environmental DNA analysis than by traditional survey, in which it is difficult to identify the species. Real-time PCR analysis using environmental DNA is an effective monitoring method that can determine both the distribution and the monthly change in biomass of *B. californica* in Korea.

## 1. Introduction

Ecologists require effective investigative tools and methods specifically designed to detect endangered species, rare species, and invasive species with low cost, minimum stress for the environment, and high potential for detection [1,2,3]. Traditional methods—such as trapping, netting, and electrofishing in field surveys—have been used routinely for the detection of various species [4]. However, these methods are unsuitable for the detection of many rare species, especially in the aquatic environment, where organisms are hard to detected visually [5,6,7]. Therefore, molecular DNA-based tools applicable for various field types of monitoring, have been developed to overcome the limitations of the traditional methods.

Environmental DNA (eDNA) analysis using species-specific molecular markers has been suggested as a method to improve the probability of monitoring [8,9]. eDNA is the term to describe biomaterials that are released into environmental materials, such as soil, freshwater, seawater, and snow [10]. The combination of species-specific molecular markers and eDNA enables identification of the species at all developmental stages, the detection of species with low abundance, and the development of an efficient and appropriate system providing early warning of invasion [11,12,13]. Recently, the application of eDNA for species detection has become increasingly common; a growing proportion of studies has used this approach, and it has been applied to various invasive taxonomic organisms—for example, amphibians [14,15,16], fishes [17,18], reptiles [19,20,21], arthropods [22], gastropods [23], bivalves [24], bryozoan [25], and hydrozoan [26].

*Bugulina californica* (Robertson, 1905), belonging to the family Bugulidae, the order Cheilostomatida, class Gymnolaemata, and phylum Bryozoa, is known as one of the fouling organisms [27]. In the aquaculture industry, fouling organisms cause problems, for example, reduced feeding rate due to overgrowth, and/or direct competition for food resources [28,29]. Arakawa (1990) [30] and Zvyagintsev (2003) [31] reported that *B. californica* competes with oyster (*Crassostrea gigas*), with an accompanying decrease in the meat content of the oyster, and formed large colonies on vessels. This species is native to the Pacific coast of North America [31], but has now been introduced to the Atlantic coast of North America, South Korea, Japan, and China [32]. In South Korea, it was first recorded in Heuksan Island in 1978, and was reported as a marine invasive species in Korea in 2010 [33,34]. It is widespread along all of coastlines of the Korean Peninsula, and negatively impacts economic activity by attaching to the facilities of the tunicate (*Halocynthia roretzi*) aquaculture farms, fishing vessels, and ship hulls [33]. It is necessary to monitor the invasive and fouling species by examination the distribution and biomass of *B. californica* in order to prepare the management. However, field survey in aquatic environment have limits, and especially class bryozoan, which requires specialized equipment such as microscope for species identification, making it difficult to do in field survey. Therefore, we have attempted to apply the eDNA method to *B. californica* monitoring.

In this study, we developed species-specific molecular markers for the rapid and accurate identification and effective monitoring of the bryozoan, *B. californica*. The newly developed species-specific markers were tested for their specificity and, *B. californica* was successfully detected in eDNA samples isolated from seawater by quantitative PCR. Through this quantitative approaching, we obtained the results for not only the presence/absence of target species, but also the amount of detected DNA to allow estimation of the biomass. We also examined the monthly changes in biomass of *B. californica*. Furthermore, to verify the efficiency of eDNA methods, we compared the eDNA methods and field survey to monitor the distribution of *B. californica*.

## 2. Materials and Methods

### 2.1. Field Survey and Specimen Morphological Examination

The field geological coordinates for this study were provided in Table 1. To improve the detection rates of *B. californica*, we conducted the field survey in summer (August, 2017) when their abundance observed in maximal condition. A total of 12 survey sites were selected based on the enough artificial substrates that various fouling organisms can attached, regular intervals, and covered three coastal.

**Figure 1:**
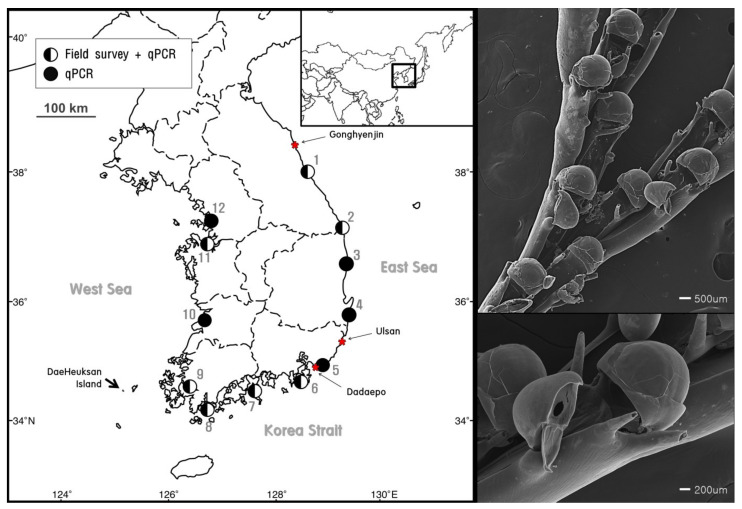
Sites of *B. californica* detection by field survey with qPCR analysis (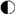) and qPCR analysis alone (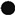). The star (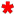) indicates the sampling sites for the molecular analysis and species analysis (**left**). Scanning electron microscope (SEM) images of *B. californica* (**right**).

To obtain the target species sample for molecular analysis and identification, *B. californica* was sampled by hand in the investigation sites (harbors) this species shown most abundant. *B. californica* samples attached on the artificial substrates, ropes, buoy, and concrete structures, were collected in February 2017 at depths ranging 0.5–5.0 m from the Gonghyeonjin (38°21′23.03″ N, 128°30′43.03″ E), Ulsan (35°31′13.01″ N, 129°22′23.06″ E), and Dadaepo (35°03′28.01″ N, 128°58′45.04″ E) harbors (Figure 1). The collected samples were immediately fixed with 95% ethanol, and stored at room temperature (25 °C). All of the colonies were examined and identified using scanning electron microscopy (SEM), and our sample images were analyzed [35,36,37]. Remaining samples were used in DNA analysis for development and validation of species-specific markers.

### 2.2. Molecular and Morphological Identification of B. californica

#### 2.2.1. DNA Extraction, Amplification, and Sequencing

Total genomic DNA was isolated from each sample of three *B. californica* sample using a DNeasy blood and tissue DNA isolation kit (QIAGEN, Hilden, Germany), in accordance with the manufacturer’s instructions. The quality and concentration of the extracted genomic DNA were determined using a Nanodrop ND-1000 spectrophotometer (Thermo Fisher Scientific, Waltham, MA, USA). All genomic DNA samples were stored at −20 °C until use.

The barcode region of the mitochondrial cytochrome c oxidase I (COI, 658 bp) gene was amplified and sequenced. The primers used for COI amplification were LCO1490 (forward) and HCO2198 (reverse) (Table 2) [38]. PCR was conducted using a 25 µL reaction mixture, containing 2.5 µL of 10× Ex Taq Buffer containing 20 mM MgCl_2_ (Clontech, CA, USA), 1 µL of 2.5 mM dNTPs (Clontech, CA, USA), 1 µL of each primer at 10 pmol, 1.5 µL of 150–250 ng/µL template DNA, 0.3 µL of 5 U/µL Taq polymerase (Clontech, CA, USA), and 17.7 µL of DEPC-treated water. The following PCR conditions were used: initial denaturation at 94 °C for 5 min; 35 cycles of denaturation at 94 °C for 30 s, annealing at 50 °C for 90 s, extension at 72 °C for 90 s; and final extension at 72 °C for 7 min. The PCR products were separated by electrophoresis on 1.5% agarose gel stained with ethidium bromide (Bioneer, Daejeon, Korea), and 2 µL of 100 bp DNA ladder (Elpis Biotech, Daejeon, Korea) was loaded to show the size of the proteins.

The PCR products were directly sequenced in both directions with the primers used for amplification (Cosmogenetech, Seoul, Korea). The results were analyzed by BioEdit [39], to assess the quality of sequencing. Three COI sequences of *B. californica* from Gonghyeonjin, Ulsan and Dadaepo were registered in GenBank of NCBI (http://www.ncbi.nlm.nih.gov accessed on 22 May 2021), and the accession numbers were MZ209214, MZ209213, and MZ209215, in respectively.

#### 2.2.2. Molecular Identification

To verify the selection of mitochondrial COI as appropriate gene region for designing the species-specific markers, we conducted the phylogenetic analysis using selected region. The sequences obtained from three *B. californica* samples were manually aligned using Clustal X [40]. Furthermore, we investigated the genetic distances and phylogenetic relationships of three Korean *B. californica* with voucher specimens of *B. californica* collected in Tongyeong (34°50′23.05″ N, 128°25′12.58″ E) registered in the Marine Bryozoans Resources Bank of Korea (resources number: MBRBK-177; GenBank accession number: MZ217204) and four other species accepted as the *Bugulina* genus, registered in the NCBI: *B. fulva* (KC129719 in UK), *Bugula flabellata* (AY633484; AY061749 in Nambia), *B. turbinata* (AY633481; AY633480; AY633482 in Canada), and *B. simplex* (AY633478 in USA). After all sequences were aligned, we identified a 507 bp region of COI in which all nucleotides overlapped. Genetic distances were calculated according to the Kimura 2-parameter (K2P) model [41], and bootstrap analysis was conducted with 1000 replicates. Phylogenetic analysis was performed using the neighbor-joining method with MEGA7.0 [42]. *Terebratalia transversa* (FJ196085 in USA), of the family Terebrataliidae, of order the Terebratulida, class Rhynchonellata, and the phylum Brachiopoda, served as outgroup for phylogenetic analyses.

### 2.3. Species-Specific Primers and eDNA

#### 2.3.1. Design and Validation of *B. californica*-Specific Primers and Probe

We designed species-specific primers amplifying short target regions [17,43]. The mitochondrial COI sequences of marine species belonging to various invertebrate taxonomic classes shared habitat with *B. californica*: Demospongiae of the phylum Porifera; Hydrozoa, Scyphozoa, and Anthozoa of the phylum Cnidaria, Ascidiacea of the phylum Chordata; Hexanauplia of the phylum Arthropoda; Crinoidea, Asteroidea, Ophiuroidea, Echinoidea, and Holothuroidea of the phylum Echinodermata; and Gymnolaemata of phylum Bryozoa were obtained from GenBank; accession numbers are presented in Table A1. These sequences were aligned using Clustal X [40], and analyzed to determine the regions conserved for *B. californica*, but sufficiently variable in related species. We selected the *B. californica*-specific nucleotide regions for primer and probe binding sites. Also, to increase the specificity for detecting target species, we designed taqman probe hybridized with only *B. californica* (Table 2).

To determine primer specificity, we used genomic DNA of species belonging to the classes of Hydrozoa, Ascidiacea, Crinoidea, Asteroidea, Ophiuroidea, Echinoidea, Holothuroidea, and Gymnolaemata (Table 3). The *B. californica*-specific region was amplified using the same PCR mixture and thermal cycling conditions used for the amplification of COI, except for the primer (species-specific primers developed in this study) and annealing temperature (60 °C). The PCR products were separated by electrophoresis on a 1.5% agarose gel stained with ethidium bromide (Bioneer, Daejeon, Korea), and 2 µL of 100 bp DNA ladder (Elpis Biotech, Daejeon, Korea) was loaded to show the size of the proteins.

The *B. californica*-specific region was amplified by qPCR using a buffer solution containing 10 µL of qPCR BIO Probe Mix Hi-ROX (PCR Biosystems, London, UK), 5 µL of template eDNA, 1 µL of TaqMan probe at 5 pmol (Metabion, Martinsried, Germany), and 1 µL of each primer at 10 pmol (Cosmogenetech, Seoul, Korea) and 2 µL of DEPC-treated water. The cycling protocol, with optimal temperatures, was 95 °C for 3 min, followed by 45 cycles at 95 °C for 5 s (denaturation) and 60 °C for 30 s (annealing/extension), using an Applied Biosystems thermal cycler (Applied Biosystems, Waltham, CA, USA).

#### 2.3.2. Collection and Isolation of eDNA

From May 2017–June 2018, 8 L of seawater was collected at the beginning of each month using a plastic beaker (2 L) in each different place, at a depth of 0.5–1.0 m, from each of 12 harbors in South Korea (Figure 1, Table 1). The seawater was first filtered through a 300 µm nylon mesh for removing large clogs and then through a 3.0 µm nitrocellulose membrane (Merck Millipore, Darmstadt, Germany) [26,27,44,45]. The membrane filters were stored in a sample tube, and the sample tubes were stored in an ice box containing dry ice (−70 °C), moved to the laboratory as soon as possible, and then processed to isolate eDNA.

eDNA was isolated from the membrane using a genomic DNA extraction kit (Bioneer, Daejeon, Korea) in accordance with the manufacturer’s instructions, but with a modifications (400 µL ddH2O was used for elution instead of the elution buffer in the kit). The quality and concentration of eDNA were determined using a Nanodrop ND-1000 spectrophotometer (Thermo Fisher Scientific, Waltham, MA, USA), and all eDNA samples were stored at −80 °C until use.

#### 2.3.3. eDNA Detection

The presence and biomass of *B. californica* DNA in the eDNA samples were analyzed by qPCR with species-specific primers and probe that were developed in this study. The probe was designed as a nucleotide sequence with high GC content and a high probability of binding (showing the low intraspecific variation or the conserved region in *B. californica*) in the sequence amplified with species-specific primers. qPCR was conducted using same qPCR mixture and thermal conditions used to confirm the sensitivity of the molecular makers with qPCR.

A *B. californica*-specific DNA fragment was used for DNA cloning (GNC Bio, Daejeon, Korea). DNA for cloning was subjected to 10-fold serial dilutions and a standard curve was plotted for quantification. We estimated the quantity of *B. californica* DNA in 8 L of seawater using a formula obtained from the y-intercept and slope, from which the number of detected DNA copies could be calculated. The number of copies of *B. californica* DNA was estimated by substituting the Ct value in the formula to estimate species biomass. PCR efficiency was calculated by substituting the slope value in the following formula: PCR efficiency (%) = ((10(−1/slope)) − 1) × 100% [46]. Also, we examined the limit of detection (LOD) and limit of quantification (LOQ) for the *B. californica* eDNA analysis as the lowest values of the linear range covered by the standard curve we established. A-cut-off values were calculated according to the method used by Kim et al. (2020) [26]. The reliable values in qPCR analysis were used as data and presented in figure.

## 3. Results

### 3.1. Molecular Assay Design

#### 3.1.1. Gene Selection for Designing Species-Specific Markers

**Figure 2:**
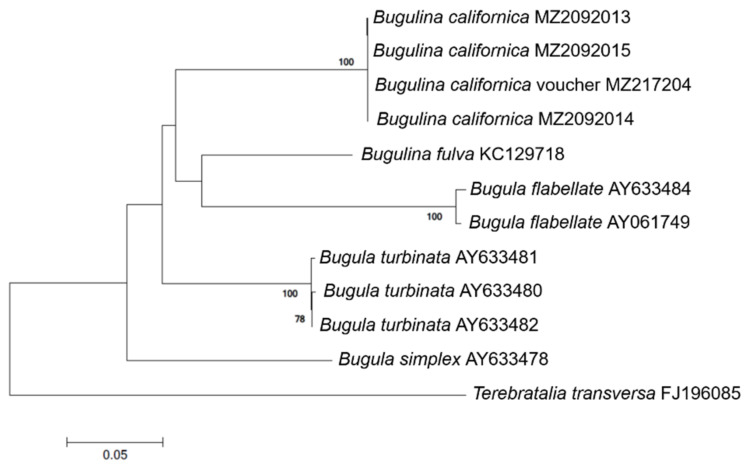
Phylogenetic tree of *B. californica* and related species constructed using 507 bp mt-COI sequences, excluding primers. GenBank accession numbers of the species are provided. Bootstrap resampling values were supported at ≥70. The scale bar indicates the genetic distances.

A 658 bp region of *B. californica* COI was successfully amplified and sequenced with universal primers, and a 507 bp region of eight COI mt-DNA sequences was obtained from GenBank. There was no intraspecific variation within *B. californica*; the interspecific variation between the other four *Bugulina* species was 18.3–25.7% (Table A2). In the phylogenetic tree, *B. californica* from Korea formed a clade with the voucher specimen of *B. californica* and was clearly distinguished from the clade of related species (Figure 2).

#### 3.1.2. Species-Specific Markers Design and Validation

**Figure 3:**
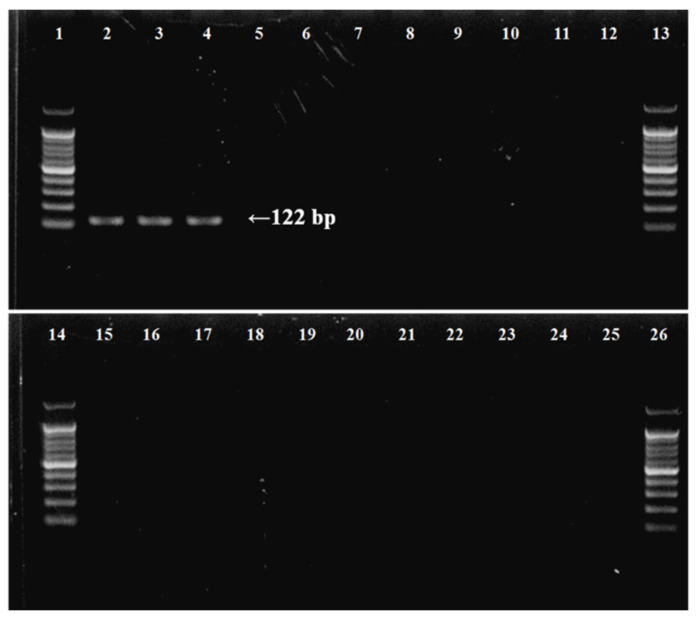
Determination of specificity of *B. californica*-specific primers by agarose gel electrophoresis of PCR products using species-specific primers for target and outgroup species. (1) 100 bp DNA ladder; (2–4) *B. californica*; (5) *Bugula neritina*; (6) *Watersipora subtorquata*; (7) *Tricellaria occidentalis*; (8) *Schizoporella unicornis*; (9) *Jellyella tuberculate*; (10) *Bougainvillia muscus*; (11) *Ectopleura crocea*; (12) *Ascidiella aspersa*; (13, 14) DNA ladder; (15) *Ciona robusta*; (16) *Antedon serrata*; (17) *Heliometra glacialis*; (18) *Patiria pectinifera*; (19) Asterias amurensis; (20) *Ophiactis savignyi*; (21) *Ophiopholis mirabilis*; (22) *Temnopleurus hardwickii*; (23) *Phalacrocidaris japonica*; (24) *Eupentacta quinquesemita*; (25) *Protankyra bidentata*; (26) 100 bp DNA ladder.

The specific primer pair was designed based on the barcode region of mitochondrial COI of *B. californica* and 138 related species (Table 2 and Table A1), namely BuCa_SF (forward), BuCa_SR (reverse), and BuCa probe (taqman probe). Designed species-specific primers within the amplified COI mtDNA region can hybridize only with the *B. californica* sequence at an annealing temperature of 60 °C, because of the differences in all the nucleotide sequences of the compared species, and the probe supported the detection of only the target species in qPCR, designed based on sequences amplified with species-specific primers. Template DNA from each species, including the various taxonomic species, were to confirm the specificity of the species-specific primer set, and a single, clear 122 bp band for each of three *B. californica* DNA samples was obtained on the agarose gel electrophoresis using the primer pair (Table 3, Figure 3). Subsequently, primer pair specificity was verified by PCR amplification for the different species, including native and non-native species inhabiting Korean coasts (Table 3). During specificity analysis of the primer pair using genomic DNA isolated from the other species, no amplification was observed (Figure 3).

**Figure 4:**
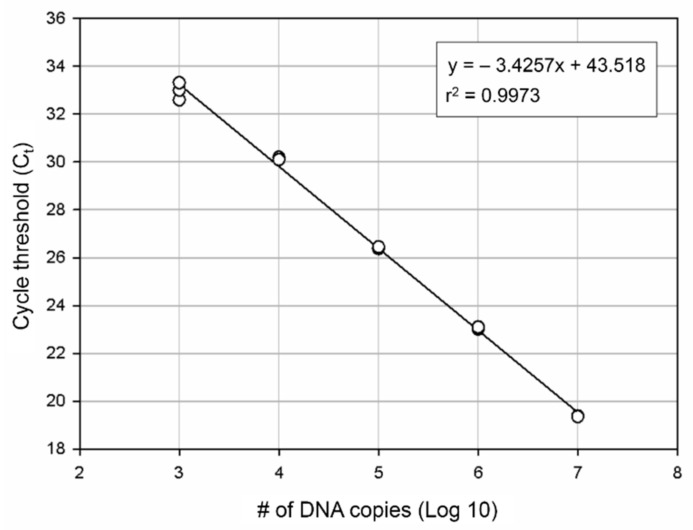
DNA quantification by qPCR. A standard curve was plotted for *B. californica*. Each point represents the C_t_ value measured for the fluorescence signal generated from serial dilutions of *B. californica* DNA. The slope is −3.4257.

For develop more accurate and efficient marine bryozoan species monitoring method, we designed the specific molecular markers for *B. californica*. We produced the standard curve for our molecular markers. The number of DNA copies was estimated by a standard curve using 10-fold plasmid dilution (Figure 4) and calculated using the formula obtained from the standard curve. The PCR efficiency calculated using the slope (−3.4257) was 96% (r2 = 0.9974).

### 3.2. Environmental DNA Monitoring

#### 3.2.1. Detection of *B. californica* in Field and eDNA Samples

Among the 12 sites investigated, *B. californica* colonies were detected in seven sites (1, 2, 6, 7, 8, 9, and 11); all of the survey sites showed amplification of *B. californica* DNA in all seawater sample replicates, whereas *B. californica* colonies were not detected at five sites (3, 4, 5, 10, and 12) using traditional survey methods (Figure 1). The Ct values for all qPCR reactions ranged from 23 to 44 and the negative control was confirmed in each experimental plate. We calculated the number of DNA copies using the y-intercept and slope after substitution of the Ct value into the formula (Figure 4).

#### 3.2.2. Calculation of *B. californica* DNA Copies

**Figure 5:**
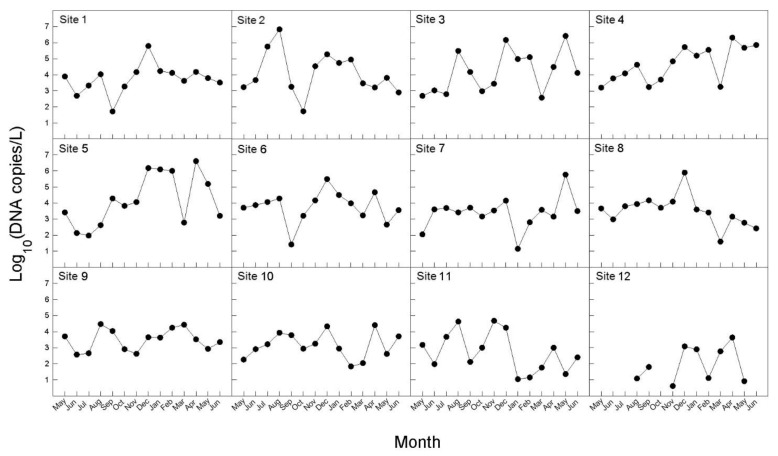
Monthly variations in eDNA concentration (copies/L) of seawater samples for *B. californica* from 12 survey sites from May 2017 to June 2018.

The concentration of *B. californica* DNA in seawater samples between May 2017 and June 2018 is shown in Figure 5. *B. californica* DNA was detected in all survey sites, except one site (12); however, Ct values were not measured for several months in site 12. In the investigational period (August–November 2017, and March–May 2018) for which DNA was detected in all of the investigated sites, the amount of DNA was lowest in site 12. We calculated the average of the number of DNA copies per month during the whole investigation period, and the range was 2274–598,117 copies/L. The months were divided in three groups, depending on the number of DNA copies (copies/L): ≤10,000 (May, June, September, October, 2017, and March, 2018); 10,000–300,000 (July, November, 2017, and January, February, June, 2018); 300,000–600,000 (August, December, 2017 and April, May 2018). In particular, in August 2017, December 2017, and April 2018, more than 500,000 copies/L of DNA were detected, which was the highest number. There was no significant difference in the total amount of DNA detected between the sea areas, and the peak was slightly different by months at the survey sites; however, seasonally, the peaks were in late summer and early autumn (August–September), early winter (November–December), and spring (March–May). The amount of the detected DNA was divided into seasons and the averages were calculated: 211,787 copies/L in spring (March–May); 181,231 copies/L in summer (June–August); 8705 copies/L in autumn (September–November); and 240,025 copies/L in winter (December–February).

## 4. Discussion

The molecular markers for the detection of the invasive bryozoan, *B. californica*, designed in this study were sensitive and accurate. The specificity of molecular markers is important for the detection of species as there may be DNA of other species in mixed samples, such as environmental samples. When developing species-specific markers, it is important to identify the target species clearly and to find a suitable region of the gene for the preparation of markers. To detect only the target species in the environmental samples, the molecular markers used should be specific and not universal. Therefore, the DNA barcode region, with a large interspecific variation and a small intraspecific variation, is appropriate for the base sequence of marker design. In this study, we selected the universal animal DNA barcoding region (658 bp of COI) to design a species-specific marker for *B. californica*. Therefore, we verified species identification by performing molecular phylogenetic analysis based on COI sequences and confirmed that samples we collected in Korea formed a monoclade with the *B. californica* voucher specimen. In addition, it was found that non-intraspecific variation and large interspecific variation (18.3–25.7%) in bryozoan COI sequences indicated an appropriate genetic site for the design of species-specific markers (Table A2).

The traditional method for species identification is based on morphological characteristics that may be affected by environmental factors, such as habitat and food, and relies on the traits of adult specimen; thus, skilled professional training and experience is required [47]. Furthermore, experts cannot often identify immature stages, juveniles, or broken specimens [13,48], and culturing the immature stages of organisms to adults for identification may be a lengthy process. These problems require a rapid and accurate new approach, such as species-specific markers that are designed based on specific nucleotides of target species. The specific genetic markers can hybridize with particular sequences only present in the target species, preventing misidentification and allowing the identification of all stages of the life cycle, even the larval stage [6].

We designed the *B. californica*-specific primers to amplify a 122 bp region of the mitochondrial COI sequence for eDNA analysis (Figure 3), and it is important to increase the possibility of target DNA detection in eDNA samples, which are degraded into short fragments by the influence of environmental factors, such as water, UV radiation, enzymes, and the activity of bacteria and fungi [49,50]. Several studies reported that eDNA is usually present as short DNA fragments (300–400 bp), and that a lake water temperature of 18 °C will maintain a 400 bp fragment of eDNA for approximately 1 week [51]. Therefore, Hajibabaei et al. (2006) [9] suggested that when detecting target DNA from degraded DNA samples, such as eDNA, designing species-specific markers that amplify only a short length are beneficial for preventing non-detection. In our study, the *B. californica*-specific primers and probe we developed were able to successfully detect the *B. californica* DNA in eDNA samples isolated from sea water (Figure 1).

We examined the distribution and abundance of *B. californica* in Korea using eDNA and compared the efficiency of eDNA analysis with the field survey. In Figure 1, more distribution sites were detected from qPCR analysis based on eDNA than field investigation that relied on visual evidence. *B. californica* DNA was detected at sites (3, 4, 5, 10, 12) where no evidence of *B. californica* was found in the field investigation. This suggested that *B. californica* is an aquatic organism that is difficult to detect by traditional methods, and showed that eDNA methods were effective for determination of the distribution of aquatic organisms [5,7] and were applicable to invasive bryozoan species in the marine environment. Therefore, our results may be due to the limitations of field survey methodologies that target aquatic organisms. However, in this study, we examined unpredictable sites using traditional methods to detect target species specific nucleotides from eDNA that was isolated from the imprints of organisms remaining in the environmental sources, without directly detecting the specimen; our data also suggested that eDNA methods can support field data and are applicable to marine environments.

In addition, we monitored monthly changes in the number of *B. californica* DNA copies detected in eDNA samples from May 2017 to June 2018 (Figure 5). In our results, the amount of DNA was slightly different at each survey site, but peaked in late summer, early winter, and spring. Maturo (1959) [52] reported that *B. californica* present at Beaufort in North America reproduced from April to early December, and that colonies were most abundant during early September and December of 1954 and late April 1955. This period appeared to be similar to the peak of amount of DNA copies showed in our results (Figure 5). Both laboratory and field studies have shown that an increase in abundance or the density of target species can lead to an increase in either eDNA concentration [16,18,45,53,54] or eDNA detectability [55]. Therefore, it can be suggested that eDNA derived from adults, rather than from larvae, appeared to have a greater effect on the results in this study. On the assumption that the adults are continuously present, if larvae, which are continuously released from late spring to early winter, had a larger effect on eDNA, larger amounts of DNA should have been detected during the release period of the larvae (larvae + adults) than in other periods (only adults). However, our results did not yield the expected results. Rather, the amount of DNA showed a difference when seasonally divided, and was highest in winter. It is expected to be the result of the seasonal difference in the water temperature affecting the maintenance of the eDNA in the environmental sample. In aquatic systems, environmental conditions influence eDNA persistence [49]. According to Strickler et al. (2015) [50], the colder the water, the greater the protection from solar radiation, and more alkaline environments are likely to hold detectable amounts of eDNA for longer than those that are warmer, sunnier, neutral, or acidic; in addition, warm water caused greater degradation of DNA by contributing to favorable environments for microbial activity. Thus, it can be supposed that the highest amount of *B. californica* DNA in winter was most likely due to greater preservation of eDNA as the water temperature is low. Nevertheless, the amount of DNA was lowest in autumn not summer. In summer, high temperature of seawater can reduce detectable eDNA templates by activating enzymes and microbial activities that lead to elevating the eDNA degradation rate. However, the *B. californica* has shown great abundance of colonies in summer, likely because *B. californica* has been reported to be more heat-resistant than other invasive bryozoan species and is less influenced by water temperature [56]. For this reason, the amount of DNA detected may not be the lowest in summer. Also, eDNA detection rate is related to the eDNA shedding rate of the target species. According to previous study, various abiotic factors—such as feeding activity, presence of co-habitat species, and temperature variation—can affect to eDNA shedding rate and degradation rate [57]. They suggested the presence of filter feeding organism as co-habitat species can cause the loss of the release DNA and affect to target species eDNA detection. Furthermore, eDNA degradation is faster in freshwater than seawater, and these showed eDNA sustained rate can increased by salinity variation with sea area [58]. However, all of these concepts were not been perfectly applied in various taxonomy and environment where many of co-habitat species exist. Each of species have different eDNA shedding rates, and there are so many types of ecosystem which have various abiotic factors and species composition. Therefore, it is expected the understating eDNA experimental study with many situation including various variables have a strong impact on future eDNA analysis.

During the investigation period in which DNA was detected in all surveyed sites, *B. californica* DNA was not detected in May−July 2017, October 2017, and June 2018 at site 12. It may be that the amount of target species DNA in environmental samples is below the threshold that can be detected using eDNA methods [15], and the causes of the absence of the *B. californica* colonies in the site 12 are thought to be invisible aquatic species and low population. The enrichment of eDNA samples may help to exceeding the threshold of detectable DNA copies, but [20] reported that altering the DNA concentration may affect PCR inhibitors and lead to false results of eDNA detection. However, if the concentration of DNA samples was low, increasing the number of PCR cycles may improve detection [59] and should be accompanied by sequencing analysis to confirm the amount and complexity of nonspecific background products [60,61].

These results suggested that the method based on the detection of DNA in environmental sources, combined with species-specific molecular markers, could be used to monitor the distribution and aspects of biomass changes as a complementary technique to traditional field survey methods.

## 5. Conclusions

In conclusion, application of environmental DNA (eDNA) method for the invasive species has invisible life stage can help the detection and monitoring. This method is efficient for the invasive bryozoan species, *B. californica*, in marine environments, as the species is difficult to detect using traditional methods, present at low densities, and at cryptic life stages, such as larva. Monitoring of the monthly changes in DNA concentration in eDNA can also be used to track increases or decreases in the abundance of the species and how various environmental factors affect the abundance of the target species, and offers advantages for managing the distribution and predicting diffusion. When the traditional survey was only method for monitor invasive bryozoan, *B. californica*, the colonies were not found in several investigation sites. On the other hand, when the molecular survey using eDNA method was applied, we can detect more *B. californica* distribution sites than colonies found sites through *B. californica* DNA detection in environmental samples. Additionally, this method make possible to examine the biomass of *B. californica* by calculation the DNA copies in eDNA detected sites. Our newly designed species-specific markers used in this study were shorter and *B. californica* specific, and it has advantage for the degraded and various species DNA mixed environmental DNA samples. Therefore, the eDNA method in this study can contribute for the supplement the limit of traditional survey, especially in case of marine invasive bryozoan species.

## Figures and Tables

**Table 1:** Geological coordinates of seawater sampling sites in the field survey.

Number	Region	Location	Latitude	Longitude
1	East Sea	Sokcho	38°12′34.76″ N	128°35′48.61″ E
2		Donghae	37°29′21.30″ N	129°07′23.35″ E
3		Jukbyeon	37°03′17.32″ N	129°25′26.30″ E
4		Yangpo	35°52′56.34″ N	129°31′35.13″ E
5	Korea Strait	Busan	35°07′02.60″ N	129°02′55.49″ E
6		Tongyeong	34°50′23.05″ N	128°25′12.58″ E
7		Yeosu	34°44′31.11″ N	127°45′20.08″ E
8		Wando	34°19′04.43″ N	126°45′11.68″ E
9	West Sea	Mokpo	34°46′51.50″ N	126°22′59.76″ E
10		Bieung	35°56′11.21″ N	126°31′38.01″ E
11		Dangjin	36°59′14.82″ N	126°44′50.86″ E
12		Incheon	37°27′34.05″ N	126°37′32.32″ E

**Table 2:** Information of primers and probe used in this study.

Primers and Probe	Sequences	Reference
LCO1490	5′-GGTCAACAAATCATAAAGATATTGG-3′	Folmer et al. 1994 [38]
HCO2198	5′-TAAACTTCAGGGTGACCAAAAAATCA-3′	
BuCa_SF	5′-CTTTTACCACCTGCACTAGCT-3′	Designed in this study
BuCa_SR	5′-GATGGTCCACTATGACCGAGA-3′	
BuCa Probe	5′-6-Fam-TGAAAGAGGAGCAGGTACAGGATGA-BHQ-1-3′	

**Table 3:** List of invertebrate species shared habitat with *B. californica* used for determination of specificity of species-specific primers.

Taxon	Location
Phylum BRYOZOA	
Class Gymnolaemata	
*Bugulina californica*	Gonghyeonjin
	Ulsan
	Dadaepo
*Bugula neritina*	Ayajin
*Watersipora subtorquata*	Jongdal
*Tricellaria occidentalis*	Kimnyeong
*Schizoporella unicornis*	Yangpo
*Jellyella tuberculata*	Seoguipo
Phylum CNIDARIA	
Class Hydrozoa	
*Ectopleura crocea*	Mulchi
*Bougainvillia ramosa*	Incheon
Phylum CHORDATA	
Class Ascidiacea	
*Ascidiella aspersa*	Chuksan
*Ciona robusta*	Yangpo
Phylum ECHINODERMATA	
Class Crinoidea	
*Antedon serrata*	Busan
*Heliometra glacialis*	Daejin
Class Asteroidea	
*Patiria pectinifera*	Juckbyeon
*Asterias amurensis*	Dadaepo
Class Ophiuroidea	
*Ophiactis savignyi*	Dodu
*Ophiopholis mirabilis*	Gampo
Class Echinoidea	
*Temnopleurus hardwickii*	Mipo
*Phalacrocidaris japonica*	Aewol
Class Holothuroidea	
*Eupentacta chronhjelmi*	Tongyeong
*Protankyra bidentata*	Incheon

## Data Availability

Not applicable.

## Appendix A

**Table A1:** Supplementary data on the design of *B. californica*-specific primers in this study.

Phylum	Class	Species	Accession Number
Porifera	Demospongiae	*Amphimedon compressa*	EU237474
		*Aplysina fulva*	EU237476
		*Callyspongia plicifera*	EU237477
		*Chondrilla nucula*	EU237478
		*Halisarca dujardini*	EU237483
		*Hippospongia lachne*	EU237484
		*Igernella notabilis*	EU237485
		*Iotrochota birotulata*	EU237486
		*Plakinastrella onkodes*	EU237487
		*Topsentia ophiraphidites*	EU237482
		*Vaceletia* sp.	EU237489
		*Xestospongia muta*	EU237490
Cnidaria	Hydrozoa	*Clava multicornis*	JN700935, NC 016465
		*Craspedacusta sowerbyi*	JN593332, NC 018537
		*Cubaia aphrodite*	JN700942, NC 016467
		*Hydra magnipapillata*	NC 011221
		*Hydra oligactis*	EU237491, NC 010214
		*Hydra sinensis*	JX089978, NC 021406
		*Hydra vulgaris*	HM369413
		*Hydra vulgaris*	HM369414
		*Laomedea flexuosa*	JN700945, NC 016463
		*Turritopsis dohrnii*	KT020766, KT899097, NC 031213
	Scyphozoa	*Aurelia aurita*	DQ787873, HQ694729, NC 008446
		*Aurelia* sp.	LC005413, LC005414
		*Cassiopea frondosa*	JN700936, NC 016466
		*Chrysaora quinquecirrha*	HQ694730, NC 020459
		*Craspedacusta sowerbyi*	JN593332
		*Haliclystus antarcticus*	KU947038, NC 030337
	Anthozoa	*Alveopora allingi*	AB907079
		*Alveopora catalai*	AB907081
		*Alveopora excelsa*	AB907085
		*Alveopora japonica*	AB907087
		*Alveopora* sp.	KJ634271
		*Alveopora spongiosa*	AB907093
		*Alveopora tizardi*	AB907096
		*Alveopora verrilliana*	AB907097
Arthropoda	Hexanauplia	*Acasta sulcata*	KJ754818, NC 029168
		*Amphibalanus amphitrite*	KF588709, NC 024525
		*Armatobalanus allium*	KJ754817, NC 029167
		*Balanus balanus*	KM660676, NC 026466
		*Capitulum mitella*	AB167462
		*Chelonibia testudinaria*	KJ754819, NC 029169
		*Chthamalus antennatus*	KP294312, NC 026730
		*Epopella plicata*	KM008743, NC 033393
		*Lepas anserifera*	KP294311, NC 026576
		*Lepas australis*	KM017964, NC 025295
		*Megabalanus ajax*	KF501046, NC 024636
		*Megabalanus volcano*	AB167539, NC 006293
		*Pollicipes mitella*	AY514042
		*Pollicipes polymerus*	AY456188, NC 005936
		*Striatobalanus amaryllis*	KF493890, NC 024526
		*Tetraclita japonica*	AB126701, NC 008974
		*Tetraclita serrata*	KJ434948, NC 029154
		*Tetraclitella divisa*	KJ754822, NC 029170
Bryozoa	Gymnolaemata	*Bugula dentata*	KC129718
		*Bugula flabellata*	AY061749
		*Bugula fulva*	KC129719
		*Bugula migottoi*	KC129720
		*Bugula neritina*	AY690838, KC129722, KC129735, KC129735, KC129754, KC129822
		*Bugula stolonifera*	KC129849
		*Bugula turrita*	KC129850
		*Celleporella hyalina*	JQ839275, JQ839276, NC 018344
		*Flustra foliacea*	JQ061319, NC 016722
		*Flustrellidra hispida*	DQ157889, NC 008192
		*Membranipora grandicella*	NC 018355
		*Tubulipora flabellaris*	EU563937
		*Watersipora subtorquata*	EU365892, NC 011820
Echinodermata	Crinoidea	*Antedon mediterranea*	AM404181, NC 010692
		*Florometra serratissima*	NC 001878
		*Neogymnocrinus richeri*	DQ068951, NC 007689
		*Phanogenia gracilis*	DQ068952, NC 007690
	Asteroidea	*Acanthaster brevispinus*	AB231476, NC 007789
		*Acanthaster planci*	AB231475, NC 007788
		*Aphelasterias japonica*	NC 025766
		*Asterias amurensis*	AB183559, NC 006665
		*Astropecten polyacanthus*	AB183560, NC 006666
		*Luidia quinaria*	AB183558
		*Patiria pectinifera*	D16387
	Ophiuroidea	*Amphipholis squamata*	FN562578, NC 013876
		*Astrospartus mediterraneus*	FN562580, NC 013878
		*Astrospartus mediterraneus*	NC 013878
		*Ophiacantha linea*	NC 023254
		*Ophiocomina nigra*	FN562577, NC 013874
		*Ophiopholis aculeata*	AF314589, NC 005334
		*Ophiura albida*	AM404180, NC 010691
		*Ophiura lutkeni*	AY184223, NC 005930
	Echinoidea	*Arbacia lixula*	NC 001770
		*Echinocardium cordatum*	NC 013881
		*Heliocidaris crassispina*	NC 023774
		*Hemicentrotus pulcherrimus*	NC 023771
		*Loxechinus albus*	JX888466
		*Mesocentrotus franciscanus*	NC 024177
		*Mesocentrotus nudus*	NC 020771
		*Nacospatangus alta*	NC 023255
		*Paracentrotus lividus*	J04815
		*Pseudocentrotus depressus*	KC490913, NC 023773
		*Sterechinus neumayeri*	NC 027063
		*Strongylocentrotus droebachiensis*	EU054306, NC 009940
		*Strongylocentrotus intermedius*	KC490912, NC 023772
		*Strongylocentrotus pallidus*	NC 009941
		*Strongylocentrotus purpuratus*	NC 001453
		*Temnopleurus hardwickii*	NC 026200
	Holothuroidea	*Apostichopus japonicus*	EU294194
		*Balanoglossus clavigerus*	NC 013877
		*Cucumaria miniata*	AY182376
		*Holothuria forskali*	NC 013884
		*Holothuria scabra*	NC 027086
		*Parastichopus californicus*	NC 026727
		*Parastichopus nigripunctatus*	NC 013432
		*Parastichopus parvimensis*	NC 029699
		*Peniagone* sp.	KF915304
		*Stichopus horrens*	HQ000092, NC 014454
Chordata	Ascidiacea	*Aplidium conicum*	FN313538, NC 013584
		*Aplidium tabarquensis*	HF548555
		*Bugulina californica*	HF548561, NC 021469
		*Botrylloides leachii*	HF548553, HG931921, NC 024103
		*Botrylloides nigrum*	HF548559, NC 021467
		*Botrylloides pizoni*	HF548554, HG931922, NC 024104
		*Botrylloides violaceus*	HF548552, NC 024256
		*Botryllus schlosseri*	FM177702, HF548550, HF548551, HG931923, NC 021463
		*Ciona intestinalis*	AJ517314, NC 004447
		*Ciona intestinalis* type *B*	AM292218, NC 017929
		*Ciona savignyi*	AB079784, NC 004570
		*Clavelina lepadiformis*	AM292603, FJ839918, NC 012887
		*Clavelina phlegraea*	AM292604, NC 024105
		*Didemnum vexillum*	KM259616, KM259617, NC 026107
		*Diplosoma listerianum*	FN313539, NC 013556
		*Halocynthia roretzi*	AB024528, NC 002177
		*Halocynthia spinosa*	HF548558, NC 021466
		*Herdmania momus*	AM292602, FN296153, NC 013561
		*Microcosmus sulcatus*	AM292321, NC 013752
		*Phallusia fumigata*	NC 009834
		*Phallusia mammillata*	AM292320, NC 009833
		*Polycarpa mytiligera*	HF548556, NC 021464
		*Pyura gangelion*	HF548557, NC 021465
		*Rhodosoma turcicum*	HF548560, NC 021468
		*Styela clava*	HG931920
		*Styela plicata*	AM292601, NC 013565

## Appendix B

**Table A2:** Pairwise distances between nucleotide sequences of mitochondrial cytochrome *c* oxidase I according to phylogenetic calculations performed using MEGA 7.0. The distances and standard errors are shown in the lower-left matrix and upper-right matrix, respectively.

		1	2	3	4	5	6	7	8	9	10	11	12
1	*Bugula flabellata* AY633484		0.004	0.022	0.026	0.022	0.022	0.022	0.025	0.025	0.025	0.025	0.038
2	*Bugula flabellata* AY061749	0.008		0.022	0.025	0.022	0.022	0.022	0.024	0.024	0.024	0.024	0.038
3	*Bugulina fulva* KC129719	0.212	0.215		0.022	0.022	0.022	0.022	0.020	0.020	0.020	0.020	0.035
4	*Bugula simplex* AY633478	0.272	0.268	0.223		0.024	0.024	0.024	0.023	0.023	0.023	0.023	0.034
5	*Bugula turbinata* AY633480	0.225	0.220	0.208	0.233		0.003	0.002	0.021	0.021	0.021	0.021	0.032
6	*Bugula turbinata* AY633481	0.223	0.217	0.211	0.233	0.004		0.002	0.021	0.021	0.021	0.021	0.033
7	*Bugula turbinata* AY633482	0.223	0.217	0.211	0.230	0.002	0.002		0.020	0.020	0.020	0.020	0.033
8	*Bugulina californica* MZ209214	0.257	0.254	0.183	0.225	0.183	0.183	0.180		0.000	0.000	0.000	0.037
9	*Bugulina californica* voucher MZ217204	0.257	0.254	0.183	0.225	0.183	0.183	0.180	0.000		0.000	0.000	0.037
10	*Bugulina californica* MZ209213	0.257	0.254	0.183	0.225	0.183	0.183	0.180	0.000	0.000		0.000	0.037
11	*Bugulina californica* MZ209214	0.257	0.254	0.183	0.225	0.183	0.183	0.180	0.000	0.000	0.000		0.037
12	*Terebratalia transversa* FJ196085	0.449	0.453	0.409	0.404	0.369	0.366	0.365	0.433	0.433	0.433	0.433	
